# Neurocognitive Targets for Psychological Assistance in Patients with the Anhedonia Phenomenon within the Framework of Affective Pathology

**DOI:** 10.1192/j.eurpsy.2024.1119

**Published:** 2024-08-27

**Authors:** A. Barkhatova, M. Popov

**Affiliations:** Department of endogenous mental disorders and affective states, Mental health research center, Moscow, Russian Federation

## Abstract

**Introduction:**

Anhedonia is a transdiagnostic psychopathological phenomenon that is considered a key feature for several disorders, primarily affective spectrum disorders. It exhibits a significant association with social and occupational maladjustment, reduced quality of life, and increased suicidal risk among psychiatric patients.

**Objectives:**

The aim of this study is to identify recommendations for psychotherapeutic assistance for patients with affective spectrum disorders.

**Methods:**

A total of 26 patients with affective spectrum disorders (ICD-10 code - F33, F31) and the phenomenon of anhedonia were examined. We utilized neuropsychological methods aimed at investigating a wide range of cognitive functions (Dynamic praxis; Color interference test; Arithmetic Tasks; Number of skips and impulsive errors; Reverse and straight rows; Verbal fluency; Design fluency; Rey-Osterritz figure) and psychometric methods designed to diagnose various types of anhedonia (consummatory (TEPS), anticipatory (TEPS), social (RSAS), and physical (PAS)).

**Results:**

Among patients with depression, the consummatory type of anhedonia was the most pronounced. A relationship was found between anticipatory anhedonia and phonetic verbal fluency (r = 0.487; p < 0.01). Additionally, there were correlations between immediate (consummatory) pleasure experience and Rey figure errors (r = -0.349; p < 0.05). Social anhedonia was associated with phonetic verbal fluency productivity (r = -0.509; p < 0.01) and performance in visual fluency productivity (r = -0.473; p < 0.01).

**Conclusions:**

The obtained results allow us to hypothesize that anhedonia is associated with difficulties both in evaluating and imagining possible positive stimuli, which leads to a lack of emotional response to the current stimulus. Thus, the availability of current pleasure may be linked to memory accessibility and regulatory function. When these domains are weakened, the respondent loses the ability to associate the current stimulus with positive past experiences, making it challenging to generate an emotional response in the current stimulus situation and disrupting the anticipation of pleasure. Based on the results, we propose the effective use of behavioral activation and work on the actualization of past experiences. Behavioral activation can be implemented by gradually introducing behaviors associated with past pleasures into the patient’s life, followed by cognitive restructuring aimed at focusing the emotional response on past and current stimuli. In addition to this, from a neurocognitive perspective, an additional element of therapy could involve training various types of cognitive functions, with an emphasis on the auditory modality.

**Disclosure of Interest:**

None Declared

